# Development, Characterization, and Evaluation of SLN-Loaded Thermoresponsive Hydrogel System of Topotecan as Biological Macromolecule for Colorectal Delivery

**DOI:** 10.1155/2021/9968602

**Published:** 2021-07-03

**Authors:** R. Xing, O. Mustapha, T. Ali, M. Rehman, S. S. Zaidi, A. Baseer, S. Batool, M. Mukhtiar, S. Shafique, M. Malik, S. Sohail, Z. Ali, F. Zahid, A. Zeb, F. Shah, A. Yousaf, F. Din

**Affiliations:** ^1^Department of Pharmacy, Beijing Shijitan Hospital, Capital Medical University, Beijing 100038, China; ^2^Beijing Key Laboratory of Bio-characteristic Profiling for Evaluation of Rational Drug Use, Beijing 100038, China; ^3^Department of Pharmaceutics, Faculty of Pharmaceutical Sciences, DOW University of Health Sciences, 74200 Karachi, Pakistan; ^4^HE.J. Research Institute of Chemistry, International Center for Chemical and Biological Sciences, University of Karachi, Karachi 75270, Pakistan; ^5^Department of Pharmacy, Abasyn University Peshawar, KPK, Pakistan; ^6^Nanomedicine Research Group, Department of Pharmacy, Faculty of Biological Sciences, Quaid-i-Azam University, Islamabad, Pakistan; ^7^Department of Pharmacy, Faculty of Medical and Health Sciences, University of Poonch Rawalakot, AJK, Pakistan; ^8^Riphah Institute of Pharmaceutical Sciences, Riphah International University, Sector G-7/4, Islamabad 44000, Pakistan; ^9^Department of Pharmacy, COMSATS University Islamabad, Lahore Campus, Lahore 54000, Pakistan

## Abstract

**Background:**

Chemotherapeutic drugs cause severe toxicities if administered unprotected, without proper targeting, and controlled release. In this study, we developed topotecan- (TPT-) loaded solid lipid nanoparticles (SLNs) for their chemotherapeutic effect against colorectal cancer. The TPT-SLNs were further incorporated into a thermoresponsive hydrogel system (TRHS) (TPT-SLNs-TRHS) to ensure control release and reduce toxicity of the drug. Microemulsion technique and cold method were, respectively, used to develop TPT-SLNs and TPT-SLNs-TRHS. Particle size, polydispersive index (PDI), and incorporation efficiency (IE) of the TPT-SLNs were determined. Similarly, gelation time, gel strength, and bioadhesive force studies of the TPT-SLNs-TRHS were performed. Additionally, *in vitro* release and pharmacokinetic and antitumour evaluations of the formulation were done.

**Results:**

TPT-SLNs have uniformly distributed particles with mean size in nanorange (174 nm) and IE of ~90%. TPT-SLNs-TRHS demonstrated suitable gelation properties upon administration into the rat's rectum. Moreover, drug release was exhibited in a control manner over an extended period of time for the incorporated TPT. Pharmacokinetic studies showed enhanced bioavailability of the TPT with improved plasma concentration and AUC. Further, it showed significantly enhanced antitumour effect in tumour-bearing mice as compared to the test formulations.

**Conclusion:**

It can be concluded that SLNs incorporated in TRHS could be a potential source of the antitumour drug delivery with better control of the drug release and no toxicity.

## 1. Introduction

Topotecan (TPT) is a water soluble compound, a synthetic derivative of camptothecin [1]. TPT possesses significant antineoplastic activity in colorectal and small cell lung cancer. It disrupts enzyme topoisomerase 1 and inhibits replication of a rapidly dividing cell. A major problem of camptothecin class drugs is their hydrolysis of lactone ring to the inactive carboxylate group at physiologic pH, decreasing the efficacy of the drug [2]. These compounds exhibit reversible pH-dependent hydrolysis at alkaline and neutral pH. There is a need to formulate a dosage form of drug maintaining its active form and an efficient sustained release. Various nanoparticle-mediated drug delivery systems are recently being investigated to check the chemical stability and improve the release profile of the antitumour drugs like TPT [1, 3]. These drug delivery systems include lipid-based nanoparticles [4], mesoporous silica nanoparticles [5], solid lipid nanoparticles (SLNs) [6], and liposomes [7]. However, most of these studies did not report the complete profile of TPT including its antitumour capability, therapeutic efficacy, and safety profile, most particularly after rectal administration. It is for this reason that we develop a new concept of drug-loaded solid lipid nanoparticle in gel for rectal delivery, which may not only enhance the therapeutic efficacy but also reduce the toxicity of the chemotherapeutic agents.

SLNs are currently focused as an alternate drug delivery system for improved access of chemotherapeutic drug to the target site [8–10]. Similarly, SLNs are reported to protect incorporated drug and offer controlled release of the drug in vivo [11–13]. An SLN-based nanocarrier system has the capability to abridge some of the drawbacks of traditional anticancer therapies, including lack of selectivity, induced tissue toxicity, reduced uptake by tumour cells, and instability [14–16]. Although being very effective for drug delivery, the SLNs sometimes demonstrate the burst release of its incorporated drugs leading to severe toxicity and failure of the therapy [17, 18]. To cope with this problem and ensure the controlled release of the incorporated drug, we incorporated the SLNs in a temperature-sensitive hydrogel to protect its immediate release. Temperature-sensitive hydrogels are prepared using poloxamer solutions (P407/P188/H_2_O) [19, 20]. These hydrogels were liquids at room temperature and gelled at body temperature [21, 22], preferred for use in injectable [23] ocular [24] and rectal [25] administration as controlled drug delivery systems. Moreover, they exhibited improved blood stream concentration of the drug in rats and humans [26].

In this study, we developed topotecan-loaded solid lipid nanoparticles (TPT-SLNs) and incorporated them in the thermoresponsive hydrogel system (TRHS) to obtain TPT-loaded SLN-mediated TRHS (TPT-SLNs-TRHS). The TPT-SLNs were a dispersion of TPT prepared by the microemulsion method, where the TRHS was a transparent hydrogel system. The SLNs and TRHS in this system (TPT-SLNs-TRHS) maintained controlled release of the TPT and prevented toxicity to the local tissues. Moreover, the TPT-SLNs-TRHS was convenient for rectal administration as it remained free flowing at below 30°C and converted to gel form at physiological conditions. It showed no signs of leakage and was well adhered to the mucosal lining of the rectum. The physicochemical and rheological behaviours, TEM, dissolution, and pharmacokinetic studies of the TPT-SLNs-TRHS were performed. Similarly, morphology and antitumour efficacy of the TPT-SLNs-TRHS were executed in xenograft nude mice.

## 2. Materials and Methods

### 2.1. Materials

Topotecan (TPT), tricaprin, and triethanolamine were purchased from Tokyo Chem. Inc. (Tokyo, Japan). Poloxamer 407 and poloxamer 188 were bought from Merk (Karachi, Pakistan). Tween 80 (polysorbate 80) was gifted by Vision Pharma Islamabad Pakistan. Span 20 was kindly gifted by Hanyang University, South Korea. The semipermeable membrane tubes were purchased from Medicell Membranes Limited (London, UK). All the other chemical reagents used in the study were utilised without any further purification.

### 2.2. Animals

Male Sprague-Dawley rats and female arrhythmic nude mice were purchased from Riphah International University, Islamabad, Pakistan. They were caged separately at 23 ± 2°C and a relative humidity of 55 ± 2% before the experiments. Water and food access was provided to the animals. Food access was stopped 12 h before the experiments. Ethical considerations for animal studies were followed during the study, especially those provided by the NIH and approved by bioethical committee Quaid-i-Azam University, Islamabad.

### 2.3. Fabrication of TPT-Loaded SLNs

TPT-SLNs were prepared by microemulsion technique with a little modification [27, 28]. A mixture of Span 20 and tricaprin was added to Tween 80 at elevated temperature, followed by addition of 1 mL distilled water under continuous stirring, until a transparent microemulsion was formed. Further, TPT was slowly mixed into the microemulsion with constant stirring. Additionally, 1 part of this hot microemulsion was then disseminated into 9 parts of cold water (2-4°C) under vigorous stirring (13,400 rpm) for 10 min, IKA Ultra-Turrax, Guangzhou, China) resulting in the preparation of SLN dispersion [1, 29]. These SLN dispersions contained 1 g of TPT and 0.5% (*w*/*v*) lipid content and stabilized by 0.5% (*w*/*v*) of surfactant and cosurfactant (Tween 80 : Span 20, 4 : 1 ratio).

### 2.4. Characterization of TPT-Loaded SLNs

#### 2.4.1. Analysis of Particle Size

TPT-SLNs were analysed for mean particle size and zeta analysis by dynamic light scattering using Zetasizer Nano ZS 90 (Malvern Instruments, Worcestershire, UK), equipped with a Helium-Neon laser that operated at a wavelength of 635 nm at 90° angle. Temperature of the systems was maintained at 25°C for analysis. A sample of 10 *μ*g TPT-SLN was dispersed in 1 mL of deionized water. It was then vortexed for 1 minute followed by the particle size analysis. Software (version 6.34, Malvern Instruments, Worcestershire, UK) was used to determine the mean particle size and distribution. The results were displayed in triplicate [30, 31].

### 2.5. Incorporation Efficiency and Total Drug Content

To determine incorporation efficiency (IE) of TPT-SLNs, 1 mL of the sample was analysed by centrifugation method. Momentarily, 1 mL of the TPT-SLN dispersion was mixed with four parts of normal saline followed by centrifugation at 24,000 rpm for 120 min at 5°C using a centrifuge machine (Eppendorf 5430 R, Hamburg, Germany). Transparent aliquot was then separated and analysed with the HPLC system. The HPLC system was comprised of ProStar 310 UV detector, ProStar 240 solvent delivery pump, and a ProStar 410 auto injector system manufactured by Varian Inc., USA. Separation was performed at 50°C utilising CromSep SS OminiSher 3 column (100 mm _ 3.0 mm, 3 lm). The mobile phase involved buffer-triethylamine (pH 5.5) and acetonitrile at volume ratio of 90 : 10 (*v*/*v*). The eluent was monitored at 381 nm with a flow rate of 0.7 mL/min and injection volume of 10 *μ*L [1, 32]. This method was modified for accurate results. The following equation was used to find out incorporation efficiency:

(1) $$\begin{array}{c} \text{Incorporation efficiency }\left(\text{IE}\%\right)=\frac{\left(W_{1}–W_{2}\right)}{W_{1}}\times 100, \end{array}$$

where “*W*_1_” and “*W*_2_” correspondingly represents weight of the total and nonentrapped drug in the SLN dispersion. Further, the following equation was used to obtain the total drug content in the SLNs:

(2) $$\begin{array}{c} \text{Drug content}\left(\%\right)=\frac{C_{1}}{C_{2}}*100 \end{array}$$

Here, *C*_1_ and *C*_2_ correspondingly represent the practical and theoretical drug concentrations.

#### 2.5.1. Fabrication of the TPT-SLNs-TRHS

TPT-SLNs-TRHS was developed by dissolving 1 g of TPT-SLN per 10 mL of poloxamer solution at 2-5°C with constant stirring. Poloxamer solution contained P 407, P 188, and distilled water at their respective weight ratio (15 : 17 g), prepared by mild stirring at 4°C. It was placed in a refrigerator overnight, until a clear TPT-SLNs-TRHS was obtained [25, 33].

#### 2.5.2. Measurement of Gelatin Temperature

Gelation temperature was measured by taking 8 g of TPT-SLN-TRHS in a transparent glass vial. A small magnetic bar was placed inside the glass vial, which was further positioned on a water bath maintained at low temperature. A digital thermometer (IKA ETS-D5, China) was inserted in the glass vial to check the temperature of TPT-SLNs-TRHS. The water bath was at constant stirring of 50-80 rpm, and its temperature was steadily increased from 25°C to 47°C. The temperature at which the magnetic bar stopped rotation was noted as gelation temperature [34].

#### 2.5.3. Gelation Time and Gel Strength

The change of the physical state of the TPT-SLNs-TRHS system from liquid form to gel was noted, and the time taken for this change was referred to as gelation time. Gel strength was the strength or viscosity of the TPT-SLNs-TRHS determined at 36.5°C. Brookfield cone and plate rheometer (DV3T, MA, USA) was used to examine the gelation time and gel strength of the TPT-SLNs-TRHS. Brookfield circulating temperature bath (TC150 MX, Middleboro, MA, USA) was used to control the temperature of the system [35].

#### 2.5.4. Measurement of Bioadhesive Force

Male Sprague-Dawley rats aged 6-8 weeks and weighing 260 ± 20 g were sacrificed, and their rectums were used for measurement of bioadhesive force. Briefly, a physical balance was used for this purpose. A small portion of the rectal tissue was placed on each of the 2 glass vials. One of them was hanged from the physical balance. The other glass vial was fixed on movable pan using adhesive tape. A drop of TPT-SLNs-TRHS was placed on the rectal tissue in the fixed glass vial. Then, the movable pan was raised till both the vials got attached. Starting from minimum, various weights were added on the other pan of the balance until both vials separated. The smallest weight that detached the vials is called bioadhesive force represented here in dyne/cm^2^ [34].

### 2.6. Transmission Electron Microscopy (TEM)

Surface morphology of TPT-loaded SLNs and TPT-SLNs-TRHS were examined using TEM (Hitachi, Japan) that operates at 100 kV. A sample of the test formulation was diluted suitably and adsorbed on carbon-coated copper grid. The surface adsorbed test formulation was negatively stained with a drop of 1% phosphotungstic acid followed by drying at room temperature [36, 37].

#### 2.6.1. Dissolution

Type 1 dissolution apparatus (VISION-6 Classic, Chatsworth, CA, USA) was used for dissolution testing of TPT-SLNs-TRHS. The results were compared to TPT solution and conventional hydrogel. Briefly, basket holding test formulation containing 20 mg of the TPT equivalent amount was dispersed in 900 mL of distilled water at 36.5°C. The apparatus was set to run at 100 rpm [25, 38]. Five milliliters of the dissolution medium was withdrawn at the designated time periods and replaced with equivalent amount of dissolution medium. The aliquot was filtered and analysed by HPLC technique as stated earlier.

#### 2.6.2. *In Vitro* Cytotoxicity Studies

In vitro cytotoxicity study of TPT solution, conventional hydrogel, blank SLNs, and TPT-SLNs-TRHS was carried out using MTT colorimetric assay. The assay was carried out in a 96-well plate by seeding previously isolated cells (at concentration of 5 × 10^3^ cells/mL) and incubated for 24 h at 37°C and 5% CO_2_ atmosphere. Then, cells were treated with a concentration range of test formulations followed by incubation at 37°C for 24 h. Afterwards, 20 *μ*L of MTT solution was added to each plate followed by incubation for 4 h at 37°C. Then, 100 *μ*L of DMSO (dimethyl sulfoxide) were added to each plate to dissolve obtained formazan crystal. Optical density (OD) was measured at 381 nm using a microplate reader [39].

The percentage cell viability was determined via the following equation:

(3) $$\begin{array}{c} \%\text{Viability}=\frac{\text{AT}-\text{AB}}{\text{AC}-\text{AB}}\times 100 \end{array}$$

where AT is the OD of the treated sample, AC is the OD of the control, and AB is the OD of the blank samples.

Percentage of viable cell was subtracted from 100 in order to obtain percent cytotoxicity.

#### 2.6.3. Pharmacokinetic Study

*(1) Administration and Blood Collection*. Pharmacokinetic studies were performed as per the previously used methods with some modifications [40, 41]. Three groups of rats with six rats in each group were categorized for animal study. After anaesthetising rats with trifluane, they were tied with thread on a surgical board in supine position. Blood samples were withdrawn from the femoral artery by inserting a polyethylene tube. One group of rats was intravenously (IV) administered with TPT solution in the left femoral vein while the other two groups were rectally administered with TPT-SLNs-TRHS system and conventional hydrogel, respectively at a dose of 10 mg/kg. Rectal administration was done with snode needle fixed on glass syringe, 4 cm above the rat anus. IV administered TPT solution acts as a control for the determination of absolute bioavailability of TPT. Periodic sampling (300 *μ*L) was obtained from the femoral artery followed by centrifugation for 10 minutes at 9000 rpm. Plasma was separated and stored at -80°C followed by drug quantification through HPLC [42].

*(2) Blood Treatment*. For the quantification of TPT, 145 *μ*L plasma was diluted with 145 *μ*L of acetonitrile [43]. Additionally, 10 *μ*L of acetonitrile solution containing 100 *μ*g/mL irinotecan was added as an internal standard. Centrifugation of the mixture was performed in order to isolate the proteins. Finally, drug content in the supernatant was analysed via HPLC.

*(3) Determination of Pharmacokinetic Parameters*. Noncompartmental analysis was performed using WinNonlin software, (Apex, NC, USA) to check various pharmacokinetic parameters in individual rats [44]. These include maximum concentration (*C*_max_), maximum time to reach *C*_max_ (*T*_max_), area under the curve (AUC) 0-infinity, and elimination constant (*K*_el_). All the results were reported as mean ± standard deviation.

### 2.7. In Vivo Antitumour Efficacy

In vivo antitumour activity was determined by a xenograft model. Tumour was introduced by subcutaneously injecting 1 × 10^6^ (100 *μ*L) cells from a cancerous cell line SCCA into the right flanks (thighs) of all mice. When the tumour volume increased to 100-150 mm^3^, its treatment was started followed by subsequent doses at a specific time period. All 24 mice were categorized into 4 groups: the three experimental and one control groups. One experimental group was treated with intravenous solution of TPT at a dose of 5 mg/kg while the other two experimental groups were rectally administered with TPT-SLNs-TRHS and conventional hydrogel, respectively. The control group was left untreated. In each mouse, tumour length and width was measured with Vernier calipers and was calculated as follows: *V* = (length × width^2^)/2. Toxicity of each formulation was determined by investigating any changes in the body weight of their respective mouse group. The antitumour effect of the rectally administered TPT-SLNs-TRHS was analysed against rectally administered conventional hydrogel and IV administered TPT solution in the tumour-induced mice by observing tumour volume and body weight change. All mice were sacrificed after the completion of experiment [45, 46].

### 2.8. Morphological Characterization

Rats were rectally administered with TPT-SLNs-TRHS and conventional hydrogel. After 24 h of dose administration, the rectum was removed and washed with normal saline followed by fixation in 10% formaldehyde. Samples were embedded in petroleum wax and sliced in thin sections (3 ~ 4 *μ*m). Morphological studies were conducted after staining with haemotoxylin and eosin and observing results in Nikkon microscope (Tokyo, Japan). Results of the untreated control group were compared with the experimental groups for changes in rectal tissues and epithelium. Some of the prominent changes seen in the experimental groups were deteriorating abrasions, epithelial shedding, atrophic changes in the mucosal membrane, and accumulation of inflammatory cells. A detailed analysis of the rectal tissue was done by calculating epithelial thickness (*μ*m) and mean mucosal changes. Analysis was performed using automated image analyser (*i*Solution FL Quebec, Canada) [47, 48].

### 2.9. Statistical Analysis

*t*-test was applied for comparison and a *P* value less than 0.05 was considered statistically significant with 95% confidence interval. Multiple comparison tests for different dose groups were conducted. Variance homogeneity was examined using the Levene test. If the Levene test indicated no significant deviations from variance homogeneity, the data were analysed by one-way ANOVA test and the least significant difference (LSD) multicomparison test. In case of significant deviations from the variance, homogeneity was observed at Levene test, a nonparametric comparison test and the Kruskal–Wallis H test were conducted. Statistical analyses were conducted using SPSS for Windows (Release 21.0, SPSS Inc., USA).

### 2.10. Stability

Stability testing of the TPT-SLNs-TRHS was conducted for 6 months as reported earlier by [49]. The test formulation was stored at 25°C and 40°C, and samples were analysed for particle size and drug content at 0-, 2-, 4-, and 6-month intervals. Moreover, physical appearance of the test formulations was observed to find any precipitation.

## 3. Results and Discussion

### 3.1. Fabrication of TPT-SLNs-TRHS

**Figure 1:**
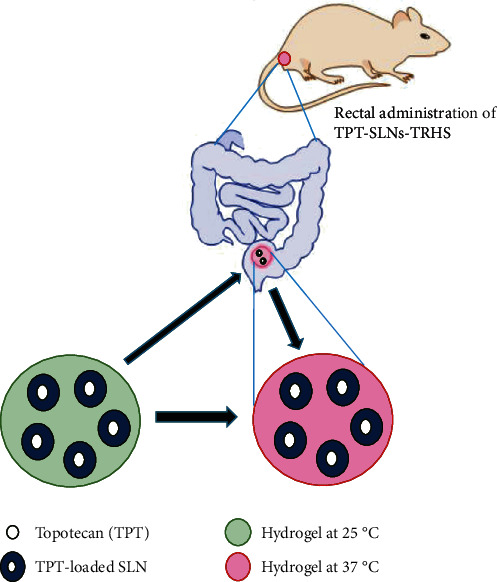
Graphical illustration of the rectal administration of TPT-SLNs-TRHS.

The TPT-SLNs-TRHS were prepared by disseminating the TPT-loaded SLNs in thermoresponsive hydrogel. This TPT-SLNs-TRHS provide twofold control release of the drug owing to its incorporation in SLNs and the thermoresponsive hydrogel system. The SLNs were composed of (TPT/lipid/surfactant/water (1 : 0.5 : 0.5 : 10, *w*/*v*)), which showed mean particle size in nanorange (about 174 nm) with excellent incorporation efficiency (90%). Tricaprin was used as the lipid for the preparation of TPT-loaded SLNs. Tween 80 and Span 20 were, respectively, used as a surfactant and cosurfactant. Their proportion was adjusted as 4 to 1. The thermoresponsive hydrogel was composed of poloxamer solution at their weight ratio of (P 407/P 188//H_2_O (15 : 17 : 58%)). The TPT-SLNs-TRHS was developed by incorporating TPT-loaded SLNs into the thermoresponsive hydrogel. The composition of the final formulation was 10 g of SLN incorporated into 32 g of poloxamer solution, which already contained 58 g double purified water. The TPT-SLNs-TRHS has gelation temperature of 31.9°C. Below this temperature, it remained a free flowing liquid whereas above this temperature, it is converted into gel (Figure 1). The SLNs and thermoresponsive hydrogel in the TPT-SLNs-TRHS was reported to overcome the erupted and quick release of the TPT, demonstrating a potential reduction in the associated toxicities. Moreover, it minimized the drug toxicity by preventing the direct contact of the TPT with rectal tissue.

### 3.2. Physicochemical Properties

#### 3.2.1. Gelation Temperature

**Figure 2:**
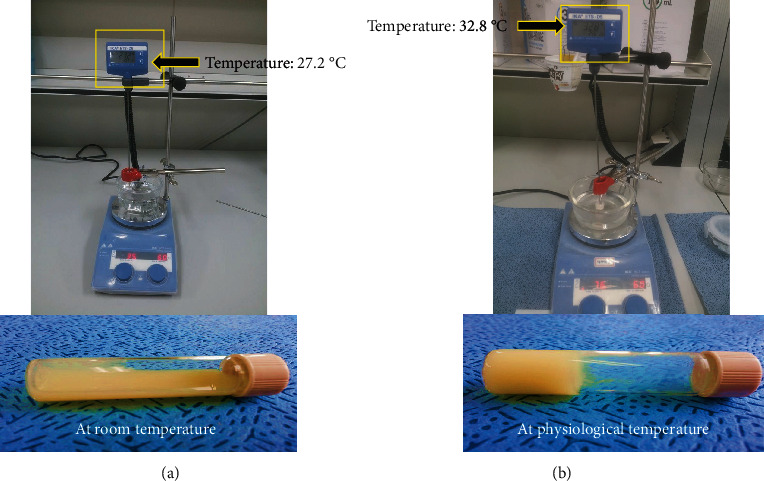
Measurement of gelation temperature at 25°C and 36.5°C. (a) The liquid behaviour at ambient temperature. (b) The gelation behaviour at physiological temperature.

**Figure 3:**
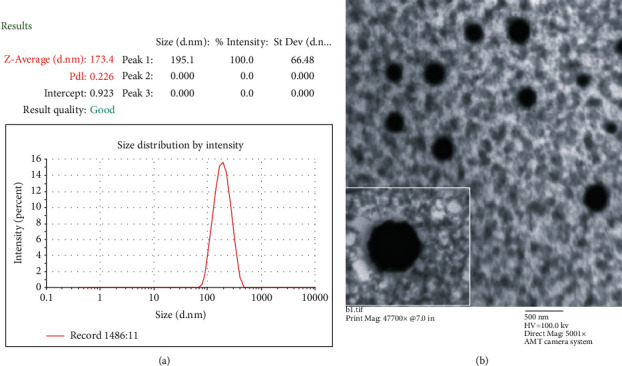
Particle characterization. (a) Mean particle size via Zetasizer. (b) Particle morphology via TEM 5001x.

It is a temperature at which a thermoresponsive system changed its liquid state into gel. One of the requirements of thermoresponsive systems is that its gelation temperature should be in the range of 30–36°C [25, 48]. However, the gelation temperature value if above body temperature may lead to a leaking problem, whereas its value below ambient may result in the formation of hard gels at room temperature, which are not required in this case. Therefore, the poloxamer mixture was chosen because of their thermoresponsive gel formation characteristics in order to develop thermoresponsive nanomicelle [50]. Additionally, poloxamers have remarkable water solubilisation effect, abridged toxicity, suitable drug release profiles, rationally low levels of skin irritation, and high solubilizing ability [51, 52]. In a preliminary study, gelation temperature of TPT-SLNs-TRHS, which is the temperature at which the liquid change to gel, was determined as 31.9 ± 0.7°C. As can be seen in Figure 2, the TPT-SLNs-TRHS was free flowing at 25°C, but it was converted into gel right after its rectal administration. This thermoresponsive behaviour of the system was credited to the poloxamer solution, as it remains liquid at ambient condition; however, quickly transformed into gel, upon the exposure to physiological temperature. The means size of TPT incorporated SLN was around 174 nm via the DLS analysis which was further confirmed by TEM image as shown in Figures 3(a) and 3(b), respectively. TEM image demonstrated nanosized particles with uniform distribution and round shape [52, 53].

#### 3.2.2. Dissolution Study

**Figure 4:**
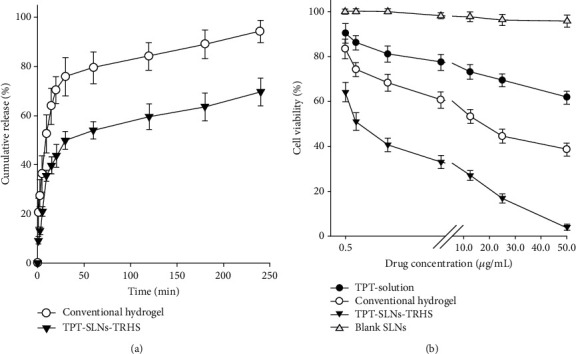
(a) Dissolution profile of drug from TPT-SLNs-TRHS as compared to conventional hydrogel. Each value represents the mean ± S.D(*n* = 3). All values in TPT-SLNs-TRHS and conventional hydrogel were meaningfully different at each time. ^∗^*P* < 0.05 as compared to conventional hydrogel. (b) In vitro cytotoxicity of blank SLNs, TPT-SLNs-TRHS, TPT solution, and conventional hydrogel after 24 h exposure in SCC-7. Data is expressed as the mean ± S.D (*n* = 8).

Figure 4(a) represents the dissolution summary of the TPT-SLNs-TRHS and conventional hydrogel. Both the test formulations were clear, owing to the solubilising effect of the poloxamer solution [19]. The TPT-SLNs-TRHS gave a meaningfully retarded dissolution rate of the TPT in association with the conventional hydrogel. An initial burst release was observed in conventional hydrogel as compared to the TPT-SLN-TRHS. Conventional hydrogel dissolves 50% of the TPT in first 5 min followed by 82% dissolution in 60 minutes; however, the TPT-SLNs-TRHS slowly dissolve TPT, as 12% of it was dissolved in 5 min, trailed by 45% in 60 minutes. Thus, the drug dissolution was remarkably reduced by the TPT-SLNs-TRHS as compared to the conventional hydrogel, resulting in reduced burst effect. This reduced drug dissolution effect of the TPT-SLNs-TRHS could be attributed to its capability of double control on the incorporated drug in the form of TPT-loaded SLNs and thermoresponsive hydrogel.

### 3.3. *In Vitro* Cytotoxicity

Cytotoxicity profiles of TPT solution, conventional hydrogel, TPT-SLNs-TRHS, and blank SLNs were evaluated to know their efficacy against cancer cells (SSC-7) as represented in Figure 4(b). The cells were incubated at 0.5, 1.0, 2.5, 5.0, 12.5, 25.0, and 50.0 *μ*g/mL drug concentrations. Blank SLNs did not display any considerable cytotoxicity for the complete range of concentrations. The cell viability persisted at more than 95% in all the cell lines even after 24 h of exposure to the formulations, indicating its biocompatible nature and tolerance [53, 54]. TPT solution demonstrated low cytotoxicity against the cancer cells as viability was reduced up to 62%; however, it was not up to the mark. This could be attributed to the cytotoxic effect of the TPT. However, since the TPT cleared quickly from the blood stream, as demonstrated in pharmacokinetic studies, thus, its cytotoxic effect was not persistent [14]. The conventional hydrogel demonstrated a significantly enhanced cytotoxicity and considerably reduced cell viability as compared to the blank SLNs and TPT solution. It could be because of their better control of the cytotoxic drug against cancer cells. The conventional hydrogel when incorporated with anticancer agents demonstrated better release properties as compared to drug solution. TPT-SLNs-TRHS showed significantly enhanced cytotoxicity as compared to blank SLNs and TPT. Further, it demonstrated significantly reduced cell viability, as almost all the cancer cells died after treatment with TPT-SLNs-TRHS. This could be because of the highly cytotoxic nature of the TPT which was potentiated by the sustained release behaviour and long-time blood circulation of the NLCs. Furthermore, the drug release was dually controlled by SLNs and TRHS which leads to enhanced cytotoxicity.

#### 3.3.1. Rectal Pharmacokinetic Study

**Figure 5:**
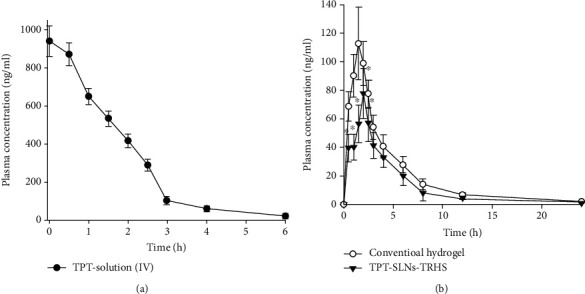
Plasma concentration vs. time profile after equivalent drug quantity administration of TPT in rats. The TPT solution was IV given whereas TPT-SLNs-TRHS and conventional hydrogel was rectally administered. Each value represents the mean ± S.D(*n* = 6).^∗^*P* was noted significantly different (<0.05) when compared with conventional hydrogel.

To obtain the pharmacokinetic profiles of the TPT-SLNs-TRHS and conventional hydrogel, they were rectally administered at a dose of 10 mg/kg, whereas TPT solution was given intravenously, at equivalent dose. The findings are represented in Figure 5 and Table 1. As expected, the TPT solution eliminated quickly from the blood flow (3-6 h) right after their IV administration exhibiting a linear pharmacokinetics (Figure 5(a)) [45, 46]. The conventional hydrogel showed an early release of drug just after the rectal administration followed by a maximum plasma concentration (112.6 ng/mL) at 1.4 h trailed by gradual decrease until 1.51 ng/mL at 24 h. However, the TPT-SLNs-TRHS achieved a maximum plasma level of about 77.69 ng/mL at 2 h and maintained a level of 1.29 *μ*g/mL at 24 h, even if the initial drug release was slower than the conventional hydrogel (Figure 5(b)). There was no significant difference between the maximum plasma concentration of conventional hydrogel and TPT-SLNs-TRHS; however, the maximum time required to reach the climax for conventional hydrogel was significantly lower than that of TPT-SLNs-TRHS which showed that burst release may occur in case of the conventional hydrogel, leading to the drug toxicity [55]. The AUC of the intravenously administered TPT solution was 1375.34 ± 139.24 ng h/mL), thus significantly higher than the AUC of TPT-SLNs-TRHS 456.23 ± 59.62 ng h/mL and hydrogel 193.69 ± 17.31 ng h/mL. However, the AUC of TPT-SLNs-TRHS was higher but not significantly different than that of conventional hydrogel. The relatively high AUC, half-life, and lower elimination rate in the TPT-SLNs-TRHS shows that the drug was released in a more retarded way as compared to the conventional hydrogel, leading to delayed drug release. Our results suggested that unlike TPT-SLNs-TRHS, a burst release may occur in TPT solution and conventional hydrogel, leading to the direct interaction of drug to body tissue which may cause tissue toxicity and side effects.

#### 3.3.2. Morphology of the Rectal Tissue

**Figure 6:**
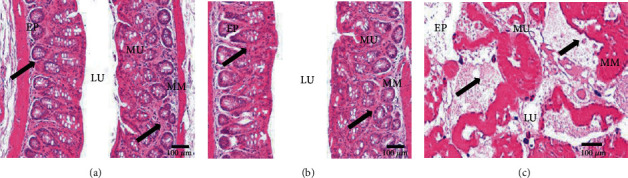
Morphology of the rectal mucosa of rats after administration of the control (a), TPT-SLNs-TRHS (b), and conventional hydrogel (c). EP: epithelium; LU: lumen; MM: muscularis mucosa; MU: mucosal layer. Scale bars = 100 *μ*m.

Rectal tissue was examined for changes in its morphology within two experimental groups treated with TPT-SLNs-TRHS and conventional hydrogel and untreated control groups (Figure 6). Generally, TPT is a known cytotoxic drug and causes cell lyses, irritation, and damage to the epithelium tissues when exposed directly. Conventional hydrogel directly released drug to rectal tissues leading to irritation and damage [55]. Rectal application of TPT-SLNs-TRHS caused no considerable change leading to irritation or damage to rectal tissue when compared with the untreated control group (Figures 6(a) and 6(b)). Moreover, no change in the thickness of the rectal epithelium or any change in the amount of mononuclear cells in lamina propria was demonstrated in the TPT-SLNs-TRHS-treated group (Table 2). It was because of the thermoresponsive nature of the TPT-SLNs-TRHS system leading to the controlled release of the drug and minimizing signs of irritation, damage, or toxicity. However, conventional hydrogel caused severe damage to the rectal tissue as evident in Figure 6(c).

#### 3.3.3. In Vivo Antitumour Ability

**Figure 7:**
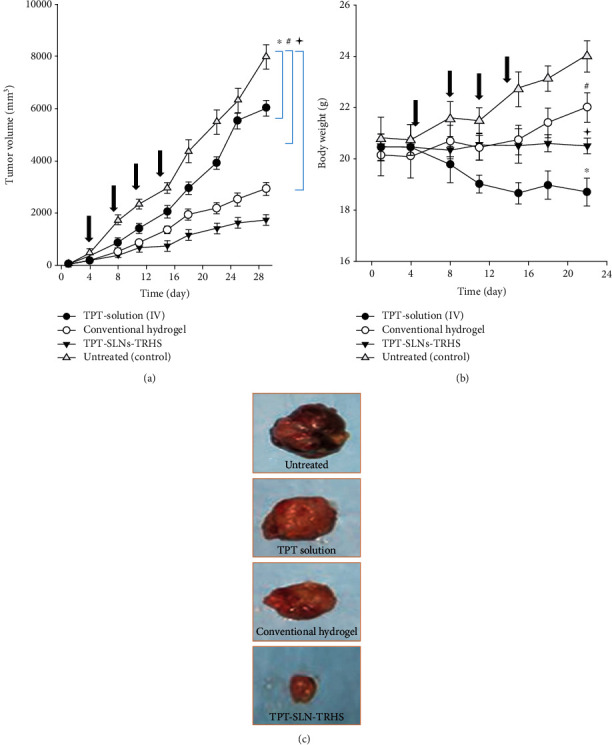
Antitumour efficacy evaluation in xenograft nude mice after application of test formulations including IV administration of TPT solution and rectal administration of TPT-SLNs-TRHS and conventional hydrogel. (a) Tumour volume analysis. (b) Body weight changes. (c) Tumour mass analysis. Each value represents the mean ± S.D (*n* = 9). ^∗^*P* < 0.05 and ^#^*P* < 0.005 as compared to the control and TPT solution; *P* < 0.001 as compared to the control, TPT solution, and conventional hydrogel.

Antitumour effectiveness of the test formulations (TPT-SLNs-TRHS, TPT solution, and conventional hydrogel) was determined, and their ability to suppress the tumour was compared with one another. The tumour expression was conducted in xenograft nude mice and assessed on the bases of tumour growth and volume. Further, the body weights of the mice were also analysed throughout the experimentation [56, 57]. As can be seen, a significant decrease in tumour cell volumes were observed in order of the TPT-SLNs-TRHS followed by conventional hydrogel and TPT solution, as compared with the untreated control group, after rectal administration of TPT-SLNs-TRHS and conventional hydrogel, and IV administration of the TPT solution. Moreover, no body weight loss or gain was perceived in TPT-SLNs-TRHS-treated mice in association to the conventional hydrogel and TPT solution-treated groups. Consequently, the antitumour effectiveness of TPT can be enhanced by using TPT-SLNs-TRHS as a drug delivery carrier, most particularly than those of conventional hydrogel and TPT solution as demonstrated in this experiment.  Figure 7 depicts the antitumour activities of the rectally administered TPT-SLNs-TRHS and conventional hydrogel and their comparison with IV administered TPT solution. The tumour growth was consistent in all the test groups after 5 days, until they received 1^st^ dose of the respective drug formulations (Figure 7(a)). The meaningfully enhanced tumour volume was observed in the control group because no treatment was provided to these mice. Unlikely, a significantly decreased tumour volume was observed in TPT solution (*P* < 0.05, 8-30 days), TPT-SLNs-TRHS, and conventional hydrogel-treated groups as compared to the control group. Moreover, the outcomes of this study indicated a significantly increased tumour volume (*P* < 0.05, 8-30 days) of the TPT solution group as compared to the TPT-SLNs-TRHS and conventional hydrogel, which can be attributed to its quick elimination from the blood stream as demonstrated in pharmacokinetics study [45, 58]. On the other hand, mice treated with TPT-SLNs-TRHS showed a significantly decreased tumour volume as compared to the conventional hydrogel.

Toxicity profiles of the test preparations were evaluated based on variation in their body weight over the extend period of study time. The TPT solution was IV administered, while the TPT-SLNs-TRHS and conventional hydrogel were given rectally. The results are demonstrated in Figure 7(b). As expected, the body weight of the control group mice was meaningfully increased as compared to the TPT-SLNs-TRHS and conventional hydrogel (*P* < 0.01, 15-18 days). Also, the TPT solution-treated group mice exhibited a substantial body weight loss when compared with the TPT-SLNs-TRHS and conventional hydrogel (*P* < 0.05, 11-22 days) and the untreated group (*P* < 0.05, 8-22 days). Furthermore, the mice treated with TPT-SLNs-TRHS and conventional hydrogel retained their body weight until the completion of study (day 22). After which, the conventional hydrogel-treated mouse weight becomes significantly different than the TPT-SLNs-TRHS (*P* < 0.05, 22 day). This could be because of the double control over the release of TPT when incorporated in TPT-SLNs-TRHS, which may prolong the release over an extended period of time leading to better antitumour effect and no toxicity. Beside this, the tumour mass was also observed and represented in Figure 7(c). The tumour mass was significantly large in the untreated control, TPT solution, and conventional hydrogel, respectively, as compared to the TPT-SLNs-TRHS. Our results suggested that the TPT-SLNs-TRHS did not induce toxicity of the loaded antitumour drug. However, the substantial body weight loss in the TPT solution and conventional hydrogel-treated mouse groups indicated severe toxicity of the loaded drug [59]. The weight gained by the untreated mouse group could be attributed to an increase in the tumour volume.

#### 3.3.4. Stability

Stability studies of the TPT-SLNs-TRHS were conducted for 6 months at ambient and physiological temperatures. The parameters used for stability analysis included physical appearance, drug content, and mean particle size. No substantial alteration was observed in the physical appearance, drug content, and mean particle size of the drug during the study duration period. Moreover, the TPT content decreased by less than 10%, even at elevated temperature of 40°C (Table 3), suggesting that TPT-SLNs-TRHS was stable for at least 6 months.

## 4. Conclusions

It is concluded that TPT was successfully incorporated into the SLN system which was further homogeneously dispersed in thermoresponsive hydrogel to develop TPT-SLNs-TRHS. This formulation was able to respond to temperature change and was suitable for rectal administration. Developed TPT-SLNs-TRHS was able to prevent initial burst release which could cause toxicity to rectum tissue and localized muscle tissues. Pharmacokinetic studies reveal that the TPT-SLNs-TRHS was suitable for sustained release effect in vivo. Stability studies exhibited that no considerable alteration in particle size and incorporation efficiency was seen over a period of six months. Further, the prepared formulation demonstrated an enhanced antitumour potential as compared to the pure drug solution. These results conclude that TPT-SLNs-TRHS is a successful candidate possessing enhanced antitumour efficacy and reduced toxicity effects.

## Figures and Tables

**Table 1:** Pharmacokinetic parameters after rectal administration of the conventional hydrogel and TPT-SLNs-TRHS and intravenous administration of TPT solution.

Parameters	TPT solution (IV)	Conventional hydrogel	TPT-SLNs-TRHS	TPT solution (IV)
AUC (ng h/mL)	1375.34 ± 139.74^∗∗^	163.69 ± 17.31	456.23 ± 59.62^∗^	1375.34 ± 139.74^∗∗^
*T*_max_(h)	—	1.43 ± 0.30	2.01 ± 0.30^∗^	—
*C*_max_ (ng/mL)	940.67 ± 80.89^∗∗^	77.69 ± 17.53	112.60 ± 25.43^∗^	940.67 ± 80.89^∗∗^

Each value represents the mean ± S.D(*n* = 6).^∗^*P* < 0.05 compared to conventional hydrogel. ^∗∗^*P* < 0.05 compared to conventional hydrogel and TPT-SLNs-TRHs.

**Table 2:** Morphological analysis of TPT-SLNs-TRHS and conventional hydrogel applied rectum.

Morphology	Control	Conventional hydrogel	TPT-SLNs-TRHS
Mucosa thickness (*μ*m)	301.42 ± 29.23	212.97 ± 16.42^∗^	298.33 ± 27.17
Epithelial thickness (*μ*m)	41.52 ± 5.92	22.61 ± 3.96^∗^	39.45 ± 4.02
Collagen percentage (%/mm^2^)	126.16 ± 29.93	175.32 ± 54.21^∗^	127.54 ± 31.58
Mononuclear cell numbers (cells/mm^2^)	43.53 ± 6.73	41.25 ± 5.94	42.73 ± 5.34

Each histological value represents the mean ± S.D(*n* = 9).^∗^*P* < 0.05 compared to the control and TPT-SLNs-TRHS.

**Table 3:** Stability test of the TPT-SLNs-TRHS at various storage conditions.

Period (months)	Particle size (nm)				Drug content (%)				Physical appearance			
	0	2	4	6	0	2	4	6	0	2	4	6
25°C	174.4 ± 12.3	176.1 ± 11.2	178.6 ± 10.4	181.5 ± 9.8	100	99.1 ± 0.2	98.2 ± 0.3	97.8 ± 0.4	Clear	Clear	Clear	Clear
40°C	174.4 ± 12.3	177.5 ± 12.3	180.7 ± 11.9	188.6 ± 9.7	100	97.0 ± 0.5	92.3 ± 0.4	90.2 ± 0.6	Clear	Clear	Cloudy	Precipitation

Each value represents the mean ± SD(*n* = 3).

## Data Availability

All the data related to this study are mentioned in the manuscript. Any further data if required may be obtained on request from the corresponding author.
